# Isolation and characterization of endophytic bacteria from tomato foliage and their in vitro efficacy against root-knot nematodes

**DOI:** 10.21307/jofnem-2021-104

**Published:** 2021-12-21

**Authors:** Binita Basumatary, Debanand Das, B. N. Choudhury, Pranab Dutta, Ashok Bhattacharyya

**Affiliations:** 1 Department of Nematology, Assam Agricultural University, Jorhat 785013, Assam, India; 2 School of Crop Protection, College of Post Graduate Studies in Agricultural Sciences, Central Agricultural University, Umiam; 3 Directorate of Research (Agri.), Assam Agricultural University, Jorhat 785013, Assam, India

**Keywords:** Culture filtrate, Efficacy, Endophytic bacteria, Exposure time, Juvenile mortality, Meloidogyne incognita, Root-knot nematode, *Solanum lycopersicum*, *Solanum pimpinellifolium*, 16S rRNA

## Abstract

Fifteen endophytic bacteria were isolated from leaves and stems of *Solanum lycopersicum* and *Solanum pimpinellifolium* collected from different locations of the Jorhat district of Assam and characterized by morphological, cultural, biochemical and molecular approaches. An in vitro study was carried out to evaluate their potentiality as biological control agents against second stage juvenile of the root-knot nematode, *Meloidogyne incognita* race2. Thirty second stage juveniles (J_2_) of *M. incognita* race 2 were exposed to cell free culture filtrates of all the 15 bacterial endophytes in a sterile cavity block at a concentration of S(100%), S/2(50%), S/4(25%), S/6(17%) and S/10(10%) for a duration of 6, 12, 24, and 48 hr. The results revealed that all the isolates had the potentiality to significantly increase the mortality of the second stage juveniles (J_2_). The percent mortality was directly proportional to the duration of exposure time and the concentration of the culture filtrate. The isolate BETL2 showed the best result with 81.47% mortality of juveniles followed by isolates BETL4 (81.43%), BETLI (79.07%), BETS2 (78.87%), and BETL6 (78.17%). The 16S rRNA sequence amplification results indicated that these isolates were *Bacillus marisflavi* (BETL2), *Bacillus altitudinis* (BETL4), *Microbacterium arborescens* (BETL1), *Exiguobacterium indicum* (BETS2), and *Bacillus marisflavi* (BETL6). The four most efficient isolates were structurally analyzed using a scanning electron microscope and this revealed that the length and breadth of isolates—BETLI, BETL2, BETL4, and BETS2 were 701.70 nm × 348.30 nm, 954.10 nm × 303.10 nm, 984.10 nm × 332.90 nm and 1422.00 nm × 742.00 nm, respectively. The result of the present study indicated that the above four novel strains of endophytic bacterial isolates enhance the mortality of J_2_ of *M. incognita* race2 and has the potentiality as biological control agents against *M. incognita*.

Endophytes are microorganisms, which colonize the living plant tissue asymptomatically, without causing any negative effect on the plant (Hirsch and Braun, 1992). Hallman et al. (1997) defined endophytes as any micro-organisms that live inside plant tissues without regard to the specific tissue colonized, and these can be isolated from surface sterilized plant tissues. The term endophytes were first introduced by de Bary in the year 1866. Later, the term was expanded to include actinomycetes, which spend part of or their whole life cycle inside the healthy living plant tissues, colonizing intra or inter-cellularly and causing no disease symptoms. Endophytic microorganisms are associated with living tissues, and may in some way contribute to the well-being of the plant (Haggag, 2010). They may facilitate their host plants to tolerate and withstand environmental stress (Malinowski and Belesky, 2000), as well as protect their hosts against pathogens and pests (Arnold et al., 2003; Akello et al., 2007) including nematodes. They have varied lifestyles and deal with the defense reactions of their hosts, overcome host resistance, enabling asymptomatic growth within the host. Endophytic bacteria colonizes an ecological niche, also used by plant pathogens, but are subjected to less competition with other microorganisms and less exposure to environmental stress factors, have sufficient supply with nutrients, and better translocation of bacterial metabolites throughout the host plant (Hallman et al., 1997). Endophytic bacteria seem to be distributed in most of the plant species and have been isolated from plant parts, namely, stems, leaves, roots, flowers, fruits and seeds (Lodewyckx et al., 2002). The population density of bacterial endophytes is higher in roots than in any other plant organ. In roots the average density is 1 × 10^5^ cfu/g fresh weight whereas averages values of 1 × 10^4^ and 1 × 10^3^ cfu/g are reported for stem and leaf, respectively (Hallmann and Berg, 2006). Abundant and diverse populations of bacterial endophytes have been identified in various crops such as potato (Sturz et al., 1999; Garbeva et al., 2001), maize (Fisher et al., 1992; McInroy & Kloepper, 1995), cotton (McInroy & Kloepper, 1995) and cucumber (Mahafee & Kloepper, 1997).

Plant parasitic nematodes are the major constraints in crop production and cause an estimated $157 billion loss annually to different agricultural crops (Abad et al., 2008). Among plant parasitic nematodes, root-knot nematodes, *Meloidogyne* spp., are recognized as the most economically important genus worldwide and its control is often dependent on the use of synthetic nematicides (Whitehead, 1998) which leads to environmental problems as well as mammalian toxicity. Control of plant parasitic nematodes by endophytes offers alternative or supplemental management tools to the use of chemicals, as endophytes negatively affect nematodes by directly repelling, attacking or killing them using toxic constituents or enzymes. The most extensively studied endophytic bacteria include *Pseudomonas* spp., *Bacillus* spp., *Serratia* spp. and *Enterobacter* spp., which all have been reported as biocontrol agents against plant parasitic nematodes (Munif et al., 2000; Vetrivelkalai et al., 2010). The potential use of endophytic bacteria isolated from cucumber, tomato and cotton such as *Aerococus viridans, Bacillus megaterium, B. subtilis, Pseudomonas chlororaphis, P. vasicularis, Serratia marcescens* and *Spingomonas pancimobilis* can reduce the population of *M. incognita* in cucumbers and tomatoes up to 50% (Hallmann, 2001). A good number of endophytic bacteria were isolated from different crop plants and are reported to control plant parasitic nematodes effectively in cotton (Hallman et al., 1997), potato (Reitz et al., 2000; Hallmann, 2001; Krechel et al., 2002;), banana (Mendoza and Sikora, 2009), and pepper (Munif and Harni, 2020). No research work has been initiated at Assam Agricultural University to isolate and evaluate the native biological control agents against nematodes. Therefore, the present study aims to isolate and characterize the native endophytic bacteria associated with the leaves and stems of the tomato plant and to evaluate their potentiality against root-knot nematode, *Meloidogyne incognita* race2.

## Materials and methods

### Collection and sterilization of plant samples

For isolation of endophytic bacteria, healthy leaf and stem samples were collected from randomly selected healthy plants of *Solanum lycopersicum* and *S. pimpinellifolium* (konbilahi), from different places of Jorhat district of Assam, namely, Alengmora, Rowriah, Malowali, Gorumora, Dergaon, and were brought to the laboratory under sterilized conditions. The collected plant samples were washed under slow running tap water to remove adhering soil particles followed by surface sterilization with sodium hypochlorite solution (2%) containing 0.1% Tween 20 for 3 min. The samples were then successively rinsed three times with sterile distilled water and, dried with sterile paper towels. All these activities were carried out under a laminar air flow cabinet (Hallman et al., 1997; Zinniel et al., 2003). For sterility check, the sterilized leaf and stem samples were placed on Nutrient agar (NA) plates as well as culturing aliquots of water from the last rinsing onto nutrient broth (NB) and kept in a BOD (Biological oxygen demand) at 28 ± 2°C for 48 hr. Samples were discarded if microbial growth was detected in the sterility check (Hallman et al., 1997; Gyaneshwar et al., 2001).

### Isolation of endophytic bacteria

The surface sterilized plant materials (stems and leaves) were dried properly in aseptic condition. The stem surface was peeled off with the help of a sterile scalpel in the laminar air flow cabinet. The stem and leave of each sample were cut into sections of 0.5 to 1 cm length and then these sections were placed on a nutrient agar medium supplemented with antifungal agents in Petri plates. The plates with plant tissues were sealed with parafilm tape and incubated at 28 ± 2°C in BOD to recover the maximum possible colonies of bacterial endophytes. After 24 to 48 hr of inoculation, morphologically different bacterial colonies were selected and were repeatedly streaked to achieve bacterial isolates (Anjum and Chandra, 2015).

### Preservation and maintenance of culture

A representative single colony of endophytic bacteria based on colony morphology was selected from the plates and transferred to slants and/or Petri plates to prepare and maintain a pure culture following standard protocols. Endophytic bacterial cultures were maintained throughout the investigation in Nutrient agar (NA) medium by routine sub-culturing at regular intervals and storage at 4°C in the refrigerator. The stored bacterial isolates were used for further studies, as well as for confirmation of results.

### Morphological and biochemical characterizations of endophytic bacteria

#### Gram staining

Gram staining was done as per the standardized protocol of Rangaswami and Bagayaraj (1993). The bacterial suspension was prepared in sterile distilled water. From this suspension a smear was prepared on a clean and dry glass slide, air dried and then heat fixed by keeping the lower side of the slide on a light flame. The smear was then flooded with 1% crystal violet solution for 1 to 2 min and then the slide was washed in clean water. After removal of excess water, Gram’s iodine stain was applied and after 30 to 60 sec, the slide was washed with clean water for few seconds and air dried. Smears on the slide were then decolorized with 95% ethyl alcohol (decolourizing agent). Decolourizing was done by holding the slide at about 45^o^ angle and adding alcohol drop by drop over the smear to flow over the smear. When the smear became colorless, the slide was washed immediately with clean water for 2 to 3 sec. Then safranin was applied to counter stain. After 45 to 60 sec, the stained slide was washed with clean water, air dried and observed under oil immersion to see the colors of the bacteria. Gram positive bacteria appear purple to blue black whereas gram negative bacteria appear red or pink. The most efficient isolates were structurally analysed using a scanning electron microscope, following the procedure of Watson et al. (1980). Biochemical tests (namely, KOH, Citrate, Gelatin hydrolysis, Catalase, Starch hydrolysis) and motility tests of all the isolates were carried out following standard procedures (Bora et al., 2015).

### Raising of pure nematodes culture

A single eggmass of *M. incognita* was collected from the pure culture maintained in brinjal at the Department of Nematology, AAU, Jorhat. After 24 hr, the hatched second stage juveniles were inoculated in pots with tomato seedlings growing in sterilized soil. These inoculated tomato plants were maintained and used as a source for inoculum for the subsequent work.

### Preparation of culture filtrates of isolated bacterial endophytes

hundred milliliters of nutrient broth media for each isolate was prepared in 250 ml Erlenmeyer flasks and seeded with isolated bacterial endophytes separately. The inoculated flasks were incubated at 28 ± 2°C on a shaker for 48 hr. The liquid culture was filtered through a Whatman No. 1 filter paper and passed through a bacterial filter (Aalten and Gowen, 1998), after which the filtrates were centrifuged at 6000 rpm for 15 min. The supernatant was collected in a 100 ml erlenmeyer flasks for further study and the suspended residues were discarded.

### In vitro bioassay against root-knot nematode juvenile

To check the efficacy of the isolated bacterial culture filtrate to kill of second stage juvenile of the root-knot nematode, *M. incognita* race2, in vitro tests were carried out. Extracted cell free culture filtrate of the isolated endophytic bacteria was considered as the stock solution—S (100% concentration). The stock solution was diluted with sterile distilled water to have S/2(50%), S/4(25%), S/6(17%) and S/10(10%) concentration of cell-free bacterial culture filtrates. Two (2) ml of cell free culture filtrate of each isolated endophytic bacteria was poured in to a sterile cavity block. To each cavity block, 30 surface sterilized *M. incognita* juveniles (J_2_) were added. All cavity blocks were kept in the laboratory at room temperature (25^o^C + 2) and arranged in a completely randomized block design (CRD) with five replications. Observation of juvenile mortality was recorded at 6, 12, 24 and 48 hr of exposure time. Apart from the treatments with different concentration of culture filtrates, sterile distilled water (SDW) was also kept as control. For determining the number of dead nematodes, a revival test was conducted by transferring the immobile juveniles to sterile distilled water and observing their activities after 24 hr under a stereo zoom binocular microscope. The juveniles showing no movement even when they were probed with bamboo splinter were considered dead. The percent juvenile mortality was calculated as follows:

$$\mathrm{Percent}\;\mathrm{mortality}=\frac{\mathrm{Number}\;\mathrm{of}\;\mathrm{dead}\;\mathrm{juveniles}\;\mathrm{in}\;\mathrm{the}\;\mathrm{treatment}}{\mathrm{Total}\;\mathrm{number}\;\mathrm{of}\;\mathrm{juveniles}\;\mathrm{in}\;\mathrm{the}\;\mathrm{treatment}}\times 100$$

### Identification of endophytic bacteria by sequencing of the 16S rRNA

The most efficient isolates in the in vitro assay were selected for amplification of the 16S rDNA. Genomic DNA was extracted using the EXpure Microbial DNA extraction kit from Bogar Bio Bee stores Pvt. Ltd. Amplification of the genomic DNA were carried out using 16S rRNA based primer i.e. forward primer—27F (5′ AGAGTTTGATCTGGCTCAG 3′) and reverse primer-1492R (5′ TACGGTACCTTGTTACGACTT 3′). For each PCR, a 25µl reaction mixture was prepared (containing Target DNA template —5 µl; Taq Master Mix—12 µl; Forward Primer —1.5 µl; Reverse Primer— 1.5 µl and Deionized water—5 µl). The PCR was performed in a thermal cycler (Applied Biosystem Pvt. Ltd.) for 22 cycles. The PCR thermal cycle consisted of an initial denaturation of 2 min at 95°C, followed 30 sec at 95°C for denaturation, 30 sec at 50°C for primer annealing and in last step primer extension for 2 min at 72°C. Steps 2, 3, and 4 were repeated for 22 cycles followed by a final extension of 10 min at 72°C.

#### Sequence analysis

The PCR products were sequenced at Triyat scientific Co. Pvt. Ltd., Maharashtra. The isolates were identified by comparative matching of the 16S rRNA gene sequence with homologus sequence using NCBI blast similarity search tool (http://www.ncbi.nlm.gov/BLAST/). The phylogeny analysis of query sequence with the closely related sequence of MUSCLE 3.7 was used for multiple alignments of sequences (Edgar, 2004) and the program Tree Dyn 198.3 was used for the construction of phylogenetic trees (Dereeper et al., 2008).

### Statistical analysis

The statistical analyses were performed by using statistical package (WASP) 2.0 of CCARI (Central Coastal Agricultural Research Institute), Goa. The percent mortality of *M. incognita* J_2_ were subjected to arc-sine transformation before analysis. The interaction effect between time, concentration and treatment were conducted by using three factorial completely randomized designs. The critical differences in main effects i.e., isolates, concentration and time of exposure as well as in their interactions were tested at *P* = 0.05. Duncan’s multiple range test (DMRT) were conducted to determine the significance difference among the treatments.

## Results

### Isolation

**Figure 1:**
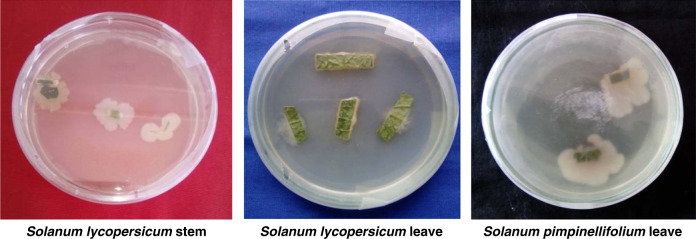
Growth of endophytic bacteria from cut pieces of stem and leaf on NA media. *Solanum lycopersicum* stem *Solanum lycopersicum* leave *Solanum pimpinellifolium* leave.

A total of 15 endophytic bacteria were isolated during the present investigation; 8 from the leaf of *Solanum lycopersicum*, 2 from the stem of *Solanum lycopersicum* and 5 from the leaf of *Solanum pimpinellifolium* (konbilahi) (Table 1) and Figure 1.

**Table 1.** Endophytic bacterial isolates from *Solanum lycopersicum* and *Solanum pimpinellifolium.*

Source	Tissue	No. of isolates	Name of isolates
*Solanum lycopersicum*	leaf	8	BETL1, BETL2, BETL3, BETL4, BETL5, BETL6, BETL7 and BETL8
*S. lycopersicum*	stem	2	BETS1 and BETS2
*Solanum pimpinellifolium* (konbilahi)	leaf	5	BEKL1, BEKL2, BEKL3, BEKL4 and BEKL5

Note: BETL1 to BETL8 = Bacteria Endophyte Tomato Leaf 1 to 8. BETS1 & BETS2 = Bacteria Endophyte Tomato Stem 1 & 2. BEKL1 to BEKL5 = Bacteria Endophyte *Kon bilahi* Leaf 1 to 5.

### Morphological and biochemical characterizations of endophytic isolates

**Figure S1:**
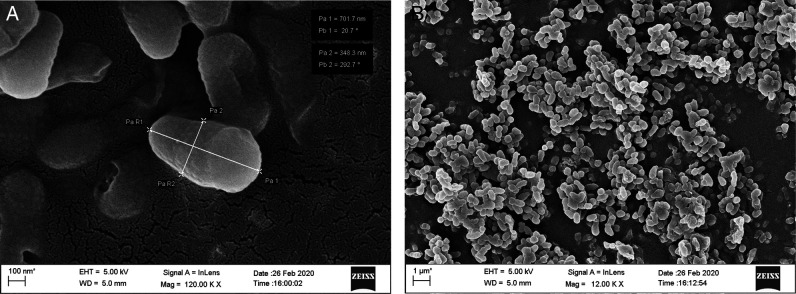
Scanning Electron Micrograph of isolate EBTL1 (*Microbacterium arborescens*).

**Figure S2:**
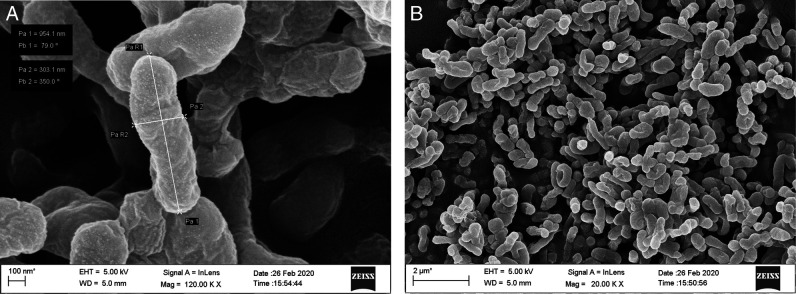
Scanning Electron Micrograph of isolate BETL2 (*Bacillus marisflavi*).

**Figure S3:**
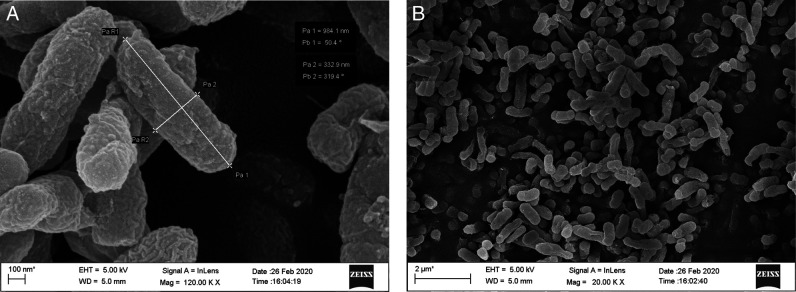
Scanning Electron Micrograph of isolate BETL4 (*Bacillus altitudinis*).

**Figure S4:**
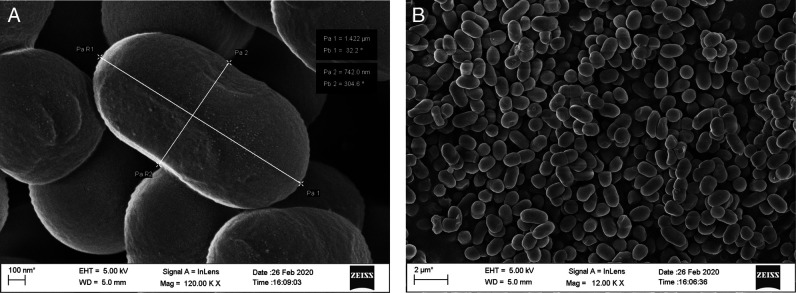
Scanning Electron Micrograph of isolate BETS2 (*Exiguobacterium indicum*).

All the isolated bacteria exhibited diverse colony, different shape and color, namely, orange, yellowish orange, shiny yellow, dark yellow, yellow, white, cream etc. Regarding cell shape and gram staining, out of the 15 isolates 6 were gram-positive rods, 4 were gram-negative rods, 2 were gram-negative diplococcus and 3 were gram-negative coccus. Among the 15 isolates, the morphological features of the four most efficient isolates based on in vitro tests were studied using Field Emission Scanning Electron Microscope (FESEM) method and are shown in Figures S1a, S1b, S2a, S2b, S3a, S3b, S4a & S4b. The biochemical tests revealed that,-9 isolates were positive for KOH, 11 isolates were positive for citrate and all the 15 isolates showed positive results for catalase and gelatine hydrolysis test. However, only 6 isolates showed positive results for the starch hydrolysis test. The result indicated that they can produce the enzyme citritase, gelatinase, catalase and starch hydrolytic enzyme. The cultural characteristics of the isolates are presented in Table 2.

**Table 2.** Morphological and biochemical characteristics of the isolated strains of endophytic bacteria.

Isolated strains	Colour	Grams’ staining	Shape	Motility	KOH	Citrate utilization	Gelatin hydrolysis	Catalase test	Starch hydrolysis
BETL1	Orange	+ve	Rod	+	-ve	+ve	+ve	+ve	-ve
BETL2	Shiny yellow	+ve	Rod	+	-ve	-ve	+ve	+ve	-ve
BETL3	Dark yellow	-ve	Rod	+	+ve	+ve	+ve	+ve	+ve
BETS1	Yellow	-ve	Rod	+	+ve	-ve	+ve	+ve	-ve
BETL4	White	+ve	Rod	+	-ve	+ve	+ve	+ve	+ve
BETL5	Yellow	-ve	Diplococcus	+	+ve	-ve	+ve	+ve	-ve
BETL6	Shiny yellow	+ve	Rod	+	-ve	+ve	+ve	+ve	-ve
BEKL1	White	-ve	Coccus	+	+ve	+ve	+ve	+ve	-ve
BETS2	Yellowish orange	+ve	Rod	+	-ve	-ve	+ve	+ve	+ve
BEKL2	Cream	-ve	Rod	+	+ve	+ve	+ve	+ve	+ve
BETL7	White	-ve	Coccus	+	+ve	+ve	+ve	+ve	+ve
BEKL3	Yellow	-ve	Diplococcus	+	+ve	+ve	+ve	+ve	-ve
BEKL4	Cream	-ve	Coccus	+	+ve	+ve	+ve	+ve	-ve
BETL8	Cream	+ve	Rod	+	-ve	+ve	+ve	+ve	+ve
BEKL5	Yellow	-ve	Rod	+	+ve	+ve	+ve	+ve	-ve

### Molecular identification

**Figure 2:**
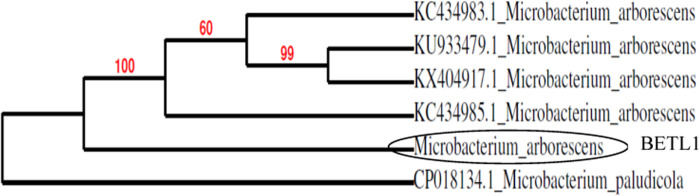
Phylogenetic tree showing the genetic relationship of the BETL1 isolate to other isolates by using maximum likelihood method.

**Figure 3:**
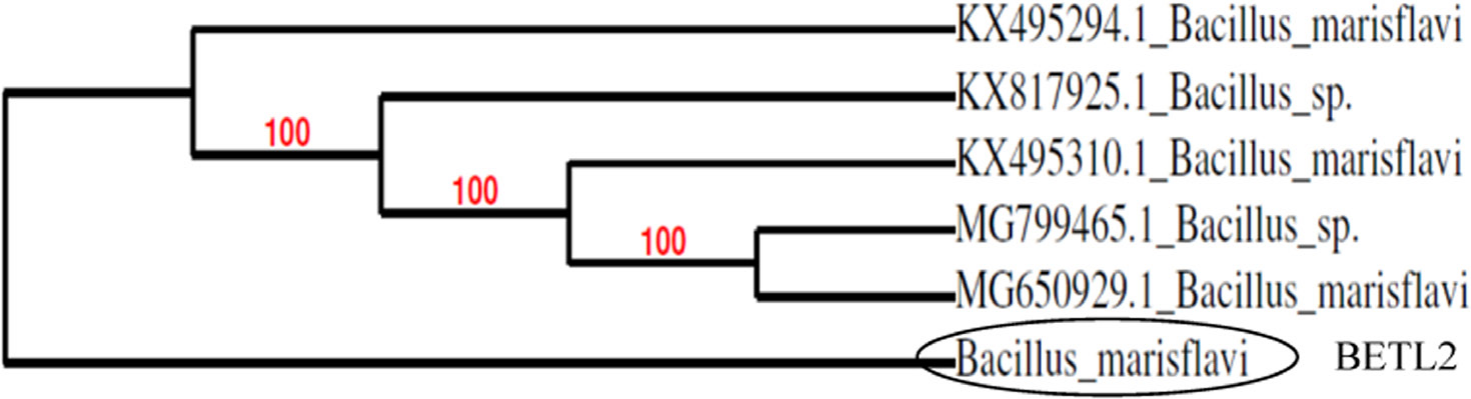
Phylogenetic tree showing the genetic relationship of the BETL2 isolate to other isolates by using maximum likelihood method.

**Figure 4:**
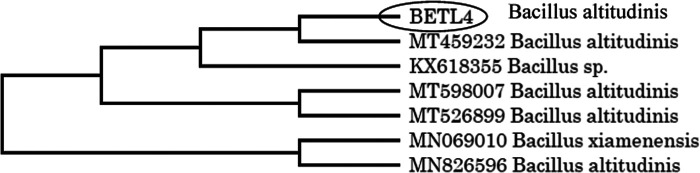
Phylogenetic tree showing the genetic relationship of the BETL4 isolate to other isolates by using maximum likelihood method.

**Figure 5:**
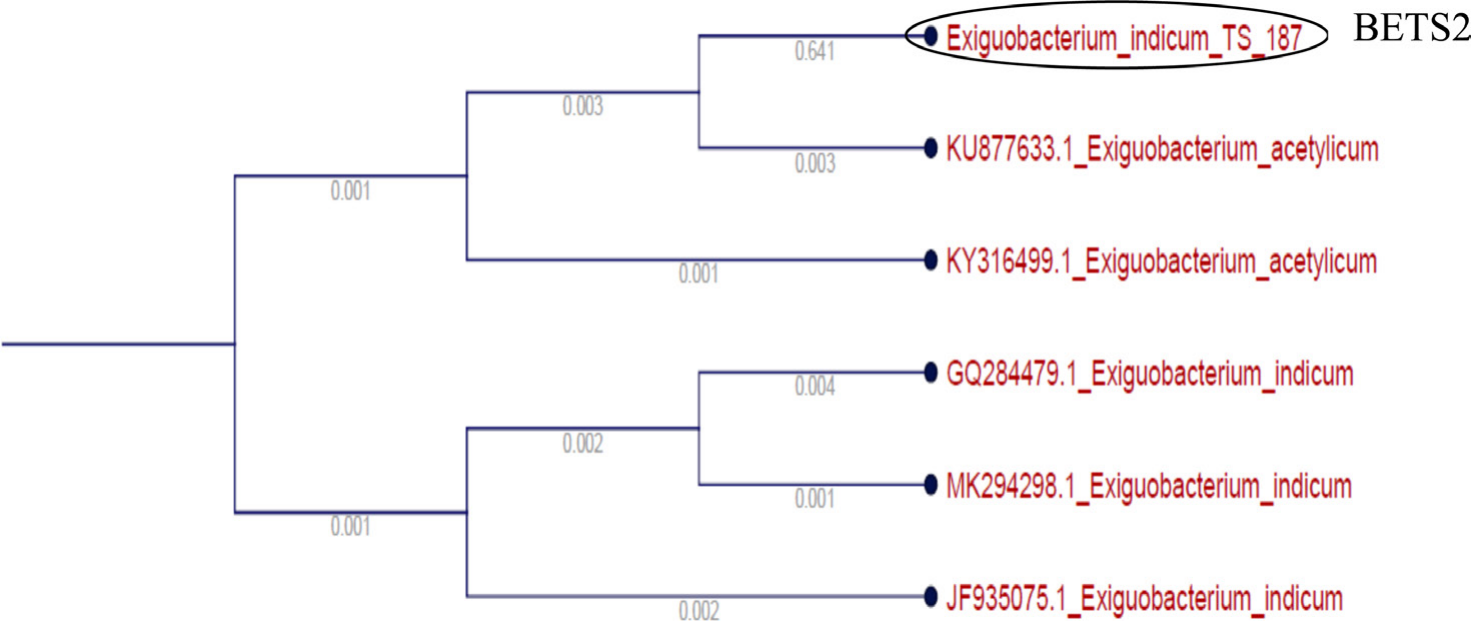
Phylogenetic tree showing the genetic relationship of the BETS2 isolate to other isolates by using maximum likelihood method.

**Figure 6:**
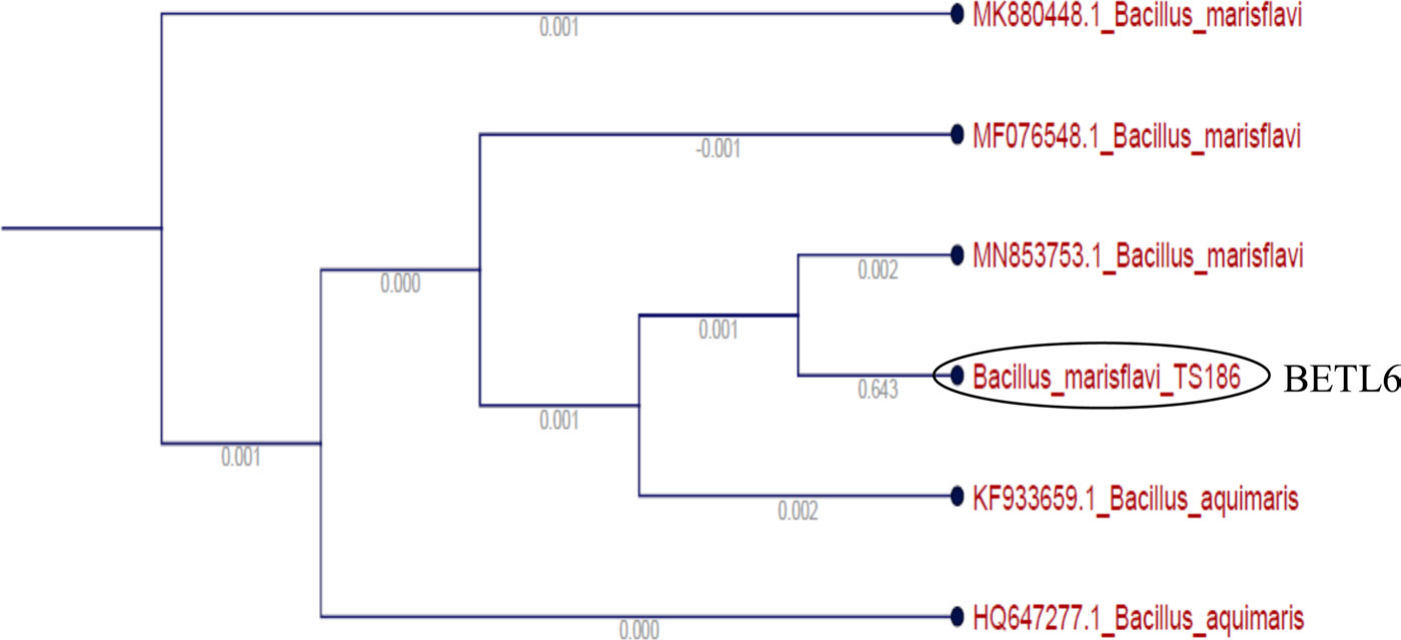
Phylogenetic tree showing the genetic relationship of the BETL6 isolate to other isolates by using maximum likelihood method.

Based on the result of in vitro mortality test against the *M. incognita* juveniles, the 5 most efficient isolates were identified by sequencing of 16S rRNA. The identified endophytic isolates were *Microbacterium arborescens* (BETL1), *Bacillus marisflavi* (BETL2), *Bacillus altitudinis* (BETL4), *Exiguobacterium indicum* (BETS2), *Bacillus marisflavi* (BETL6) and are shown in Figures 2–6.

### In vitro bioassay against root-knot nematode

**Figure 7:**
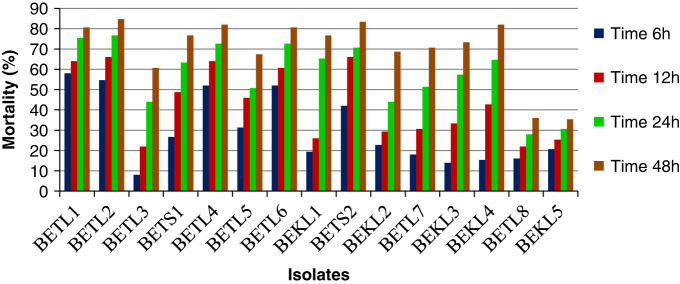
Effect of culture filtrates of isolated bacterial endophytes on juvenile mortality of *Meloidogyne incognita* at 10% (S/10) concentration at different exposure time.

The culture filtrates of all isolated bacterial strains exhibited varying degrees of nematicidal effect on second stage juvenile (J_2_) of *M. incognita* under laboratory conditions and were significantly different from control (Table 3). The percent mortality of J_2_ of *M. incognita* was found to be directly proportional to the concentration of the culture filtrate and the period of exposure time (Table 3 and Fig. 7). All the fifteen isolated bacterial endophytes showed significant increase in mortality of J_2_ of *M. incognita* irrespective of concentrations of the culture filtrates (C) and duration of exposure time (t) as compared to the control (SDW), where no mortality of J_2_ of *M. incognita* was recorded. Among the 15 strains of isolated endophytes, the culture filtrate of BETL2 (*Bacillus marisflavi*) was found to cause maximum mortality of J_2_ of *M. incognita* at all the tested concentrations compared to other endophytes, with 100% mortality of J_2_ of *M. incognita* in S and S/2 concentration and 84.67% mortality in S/10 concentration after 48 hr of exposure time, followed by BETL4 (*Bacillus altitudinis*) and BETL1 (*Microbacterium arborescens*). Irrespective of concentration of culture filtrate and period of exposure time, the mortality rate of J_2_ of *M. incognita* caused by isolate BETL2 reached 81.47%, which was at par with isolate BETL4 (81.43%), followed by isolate BETL1 (79.07%), which is at par with isolate BETS2 (78.87%) followed by BETL6 (78.17%). There was no significant difference between BETS2 and BETL6. All the fifteen isolates, except BETL8 and BEKL5 caused more than 44% J_2_ mortality during 24 hr exposure time at S/10 concentration of culture filtrate (Fig. 7). Irrespective of isolate and exposure time, the mortality rate of *M. incognita* J_2_, at the lowest concentration (S/10) was 47.24% whereas, at the highest concentration (S), mortality rate was 76.51%. Similarly, irrespective of isolate and concentration of culture filtrate, the mortality rate of *M. incognita* J_2_ was highest 76.23% at 48 hr exposure time and lowest 46.93% at 6 hr exposure time (Table 3).

**Table 3.** Effect of culture filtrate of isolated bacterial endophytes on juvenile mortality of *Meloidogyne incognita.*

		Period of exposure (hrs)					
Treatment	Culture filtrate concentration	6	12	24	48	Treatment (T) Mean	Culture filtrate concentration (C) Mean
BETL1	S/10	58.00 (49.61)	64.00 (53.18)	75.33 (60.23)	80.67 (63.96)	79.07 (63.64)^c^	47.24 (42.36)
	S/6	66.67 (54.86)	71.33 (57.67)	78.00 (62.04)	84.00 (66.48)		55.78 (47.70)
	S/4	72.00 (58.09)	78.67 (62.51)	80.00 (63.49)	88.67 (70.53)		63.09 (52.62)
	S/2	73.33 (61.60)	82.00 (64.96)	84.67 (66.97)	92.00 (73.94)		69.79 (57.62)
	S	81.33 (64.49)	86.67 (68.70)	88.67 (70.53)	95.33 (78.90)		76.51 (63.12)
BETL2	S/10	54.67 (47.68)	66.00 (54.34)	76.67 (61.16)	84.67 (67.08)	81.47 (66.74)^a^	
	S/6	60.00 (50.79)	72.00 (58.09)	80.67 (64.05)	90.00 (71.76)		
	S/4	64.00 (53.14)	78.00 (62.04)	85.33 (67.61)	94.00 (76.11)		
	S/2	78.00 (62.23)	85.33 (67.61)	91.33 (72.95)	100.00 (89.93)		
	S	83.33 (65.99)	89.33 (71.06)	95.33 (78.90)	100.00 (89.93)		
BETL3	S/10	8.00 (16.35)	22.00 (27.90)	44.00 (41.52)	60.67 (51.19)	49.07 (44.17)^m^	
	S/6	10.67 (18.93)	28.00 (31.78)	47.33 (43.47)	64.00 (53.14)		
	S/4	19.33 (26.04)	38.00 (37.99)	60.67 (51.18)	70.67 (57.25)		
	S/2	26.00 (30.63)	52.67 (46.53)	67.33 (55.15)	78.00 (62.09)		
	S	50.00 (45.00)	67.33 (55.28)	80.67 (63.93)	86.00 (68.11)		
BETS1	S/10	26.67 (31.04)	48.67 (44.23)	63.33 (52.75)	76.67 (61.23)	65.90 (55.13)^jk^	
	S/6	31.33 (33.98)	52.00 (46.15)	72.00 (58.13)	80.00 (63.49)		
	S/4	35.33 (36.45)	62.67 (52.36)	78.00 (62.09)	82.67 (65.45)		
	S/2	40.00 (39.22)	69.33 (56.41)	80.00 (63.49)	88.67 (70.63)		
	S	67.33 (55.15)	80.67 (63.96)	89.33 (71.06)	93.33 (75.23)		
BETL4	S/10	52.00 (46.15)	64.00 (53.14)	72.67 (58.50)	82.00 (64.96)	81.43 (66.62)^ab^	
	S/6	61.33 (51.59)	72.67 (58.50)	80.00 (63.46)	86.00 (68.11)		
	S/4	70.00 (56.85)	79.33 (63.00)	87.33 (69.24)	91.33 (72.95)		
	S/2	76.67 (61.27)	85.33 (67.51)	93.33 (75.23)	97.33 (82.77)		
	S	86.67 (68.70)	91.33 (72.95)	100.00 (89.93)	100.00 (89.93)		
BETL5	S/10	31.33 (34.01)	46.00 (42.69)	50.67 (45.38)	67.33 (55.21)	64.20 (53.72)^l^	
	S/6	40.00 (39.20)	57.33 (49.22)	66.00 (54.42)	70.67 (57.39)		
	S/4	46.67 (43.06)	66.00 (54.34)	72.67 (58.50)	77.33 (61.60)		
	S/2	48.67 (44.23)	72.67 (58.50)	78.00 (62.09)	82.00 (64.99)		
	S	62.00 (51.96)	78.00 (62.14)	82.67 (65.45)	88.00 (69.93)		
BETL6	S/10	52.00 (46.15)	60.67.00 (51.21)	72.67 (58.55)	80.67 (63.96)	78.17 (63.56)^de^	
	S/6	60.67 (51.17)	70.00 (56.80)	78.00 (62.09)	84.00 (66.48)		
	S/4	66 (54.39)	76.67 (61.16)	82.67 (65.45)	89.33 (71.06)		
	S/2	72.00 (58.14)	80.00 (63.46)	88.00 (69.93)	94.67 (78.21)		
	S	78.67 (62.56)	86.00 (68.11)	90.67 (72.36)	100.00 (89.93)		
BEKL1	S/10	19.33 (25.92)	26.00 (30.54)	65.33 (53.97)	76.67 (61.18)	67.20 (56.23)^h^	
	S/6	36.00 (36.84)	42.67 (40.78)	74.67 (59.83)	80.67 (63.99)		
	S/4	44.00 (41.52)	48.67 (44.23)	80.00 (63.53)	84.67 (67.08)		
	S/2	65.33 (53.97)	76.00 (60.69)	82.67 (65.58)	89.33 (71.06)		
	S	81.33 (64.45)	87.33 (69.24)	88.67 (70.53)	94.67 (79.60)		
BETS2	S/10	42.00 (40.39)	66.00 (54.36)	70.67 (57.27)	83.33 (65.94)	78.87 (65.15)^cd^	
	S/6	51.33 (45.76)	71.33 (57.68)	80.67 (64.05)	87.33 (69.50)		
	S/4	60.00 (50.78)	80.67 (63.96)	86.00 (68.11)	90.00 (71.76)		
	S/2	70.67 (57.27)	84.00 (66.48)	90.67 (72.36)	96.67 (81.88)		
	S	82.67 (65.45)	88.67 (70.53)	94.67 (78.21)	100.00 (89.93)		
BEKL2	S/10	22.67 (28.30)	29.33 (32.77)	44.00 (41.55)	68.67 (55.99)	73.33 (60.72)^f^	
	S/6	60.00 (50.80)	69.33 (56.45)	79.33 (62.99)	85.33 (67.57)		
	S/4	68.67 (55.99)	76.00 (60.74)	84.00 (66.48)	89.33 (71.42)		
	S/2	72.00 (58.09)	80.67 (63.96)	88.67 (70.47)	94.67 (78.21)		
	S	77.33 (61.65)	84.00 (66.48)	92.67 (74.53)	100.00 (89.93)		
BETL7	S/10	18.00 (25.01)	30.67 (33.61)	51.33 (45.77)	70.67 (57.28)	66.23 (55.37)^j^	
	S/6	27.33 (31.47)	41.33 (39.99)	61.33 (51.57)	80.67 (64.05)		
	S/4	54.00 (47.31)	70.00 (56.85)	76.67 (61.14)	85.33 (67.51)		
	S/2	68.00 (55.56)	75.33 (60.25)	83.33 (65.94)	91.33 (72.95)		
	S	76.00 (60.69)	81.33 (64.49)	87.33 (69.24)	94.66 (76.81)		
BEKL3	S/10	14.00 (21.89)	33.33 (35.24)	57.33 (49.23)	73.33 (58.99)	67.19 (56.08)^hi^	
	S/6	36.00 (36.86)	46.67 (43.08)	71.33 (57.67)	79.33 (63.15)		
	S/4	52.00 (46.15)	61.33 (51.61)	82.00 (64.99)	86.67 (68.70)		
	S/2	66.00 (54.34)	73.33 (58.94)	86.00 (68.37)	90.67 (72.36)		
	S	70.67 (57.23)	80.00 (63.46)	89.33 (71.06)	94.67 (78.21)		
BEKL4	S/10	15.33 (22.92)	42.67 (40.78)	64.67 (53.55)	82.00 (64.99)	71.33 (59.95)^g^	
	S/6	20.67 (26.94)	54.67 (47.68)	78.00 (62.09)	87.33 (69.24)		
	S/4	34.00 (35.61)	66.67 (54.79)	88.00 (69.93)	92.67 (74.53)		
	S/2	66.00 (54.36)	80.00 (63.46)	91.33 (73.24)	96.67 (80.67)		
	S	82.67 (65.49)	88.67 (70.53)	94.67 (78.21)	100.00 (89.93)		
BETL8	S/10	16.00 (23.51)	22.00 (27.90)	28.00 (31.91)	36.00 (36.86)	37.07 (37.25)^o^	
	S/6	21.33 (27.46)	26.00 (30.63)	32.67 (34.84)	42.67 (40.78)		
	S/4	27.33 (31.49)	31.33 (34.03)	38.00 (38.04)	49.33 (44.62)		
	S/2	32.67 (34.84)	36.67 (37.26)	46.67 (43.08)	54.00 (47.31)		
	S	40.00 (39.22)	46.00 (42.70)	52.67 (46.53)	62.00 (52.01)		
BEKL5	S/10	20.67 (27.03)	25.33 (30.19)	30.67 (33.61)	35.33 (36.46)	39.20 (38.61)^n^	
	S/6	26.67 (31.07)	30.67 (33.61)	36.00 (36.84)	40.00 (39.22)		
	S/4	30.00 (33.19)	35.33 (36.46)	42.67 (40.77)	46.67 (43.08)		
	S/2	35.33 (36.46)	42.00 (40.39)	48.67 (44.23)	52.67 (46.53)		
	S	41.33 (40.01)	49.33 (44.62)	55.33 (48.07)	59.33 (50.39)		
Distilled Water (SDW)	S	0	0	0	0	(0.00)	
	S	0	0	0	0		
	S	0	0	0	0		
	S	0	0	0	0		
	S	0	0	0	0		
Period of Exposure(t) Mean		46.93 (42.16)	58.28 (49.34)	68.51 (56.29)	76.23 (62.95)		

Note: CV = 5.50, CD(P = 0.05): Treatment (T): 0.80; Concentration (C): 0.45; Period of exposure (t): 0.40; T × C: 1.79; T×t: 1.61; C×t: 0.89; T × C×t: 3.59. Figures in the parentheses are Arc-Sine transformed values. Mean followed by the same letter in the superscript(s) are not significantly different.

## Discussion

During the present study, fifteen endophytic bacteria were isolated from leaves and stems of *Solanum lycopersicum* and *S. pimpinellifolium* and these were characterised for their physical, cultural and biochemical properties. Endophytic bacteria can be isolated from diverse plant species, grown under different ecological conditions (Zinniel et al., 2002; Nair and Padmavathy, 2014) and from both above and below ground plant parts; roots being the major source of these bacteria as compared to above ground parts (Rosenblueth and Martinez-Romero, 2006; Senthilkumar et al., 2011). Nawangsih et al. (2011) isolated 49 endophytic bacteria from healthy tomato stems while, Yang et al. (2011) isolated 72 endophytic bacteria from tomato, of which 27 strains were isolated from leaves and 45 strains were isolated from stems. Amaresan et al. (2012), Purnawati et al. (2014) and Abdallah et al. (2018) isolated 87, 10, and 38 endophytic bacteria, respectively from different parts of tomato plants. Sai Sushma et al. (2002) also isolated 24 endophytic bacteria from different plant tissues including root, stem and fresh leaves of tomato plants. These findings of isolation of endophytic bacteria from tomato leaves and stems were in agreement with our present findings.

The preliminary identification of the isolated endophytic bacteria was done based on morphological, cultural and biochemical features. Among the 15 isolates, there were gram-positive rods, and gram-negative rods, diplococcus and coccus. In the biochemical tests, 9 isolates were positive to KOH, 11 isolates were positive to citrate and all the 15 isolates showed positive results for catalase and gelatine hydrolysis test however, only 6 isolates showed the positive results for starch hydrolysis test. Similar results have been found in previous studies. Patel et al. (2012) isolated eighteen (HR1 to HR18) endophytic bacteria from the stem and roots of tomato plant from different region of Gujarat, India and found that, 10 bacteria (55.55%) were gram-negative coccobacilli, 5 bacteria (27.77%) gram-positive bacilli and 3 bacteria (16.66%) were gram-negative cocci. Prasom et al. (2017) isolated 43 endophytic bacteria from healthy tomato plants and found that 79.07% bacteria were gram-positive whereas, 20.93% were gram-negative bacteria with different colony characteristics; of these, 79.% bacteria showed KOH positive test, 20.93% showed KOH negative test; 27.91% bacteria were coccus shaped, 60.46% were bacillus and only 11.63% were coccobacilli in shape.

The five most effective endophytic bacteria against second stage juveniles of *Meloidogyne incognita* were identified using molecular tool as *Bacillus marisflavi* (two strains), *Bacillus altitudinis, Microbacterium arborescens* and *Exiguobacterium indicum*. Chaturvedi et al. (2016) and Hallman et al. (1997) reported that *Bacillus, Burkholderia, Microbacterium, Micrococcus, Pantoea, Pseudomonas* and *Stenotrophomonas* were the most commonly isolated genera, of which *Bacillus* and *Pseudomonas* are the predominant genera. The statement is in agreement with the present findings. Nandhini et al. (2012) also isolated several endophytic bacteria from leaves, stem, root and fruits of tomato plants and identified their genera as *Bacillus, Pseudomonas, Klebsiella* and *Citrobacter*. Espinoza et al. (2020) isolated 25 endophytic bacteria from tomato and molecularly identified as *Methylobacterium radiotolerans, Shinella* sp.*, Burkholderia cepacia, Sphingobium herbicidovorans, Pseudomonas* sp*., Achromobacter xylosoxidans* and *Rhizobium radiobacte*. It indicated that diverse genera of endophytic bacteria could be isolated from tomato plants.

In the present study cell free culture filtrates of all fifteen bacterial isolates exhibited nematicidal property in vitro against J_2_ of *M. incognita*; *B. marisflavi* being the best. Similar findings have been reported previously. There was a substantial mortality of second stage juveniles of *Meloidogyne incognita* when exposed to the cell free culture filtrate of *Bacillus subtilis* under in vitro condition (Wei et al., 2014), and the mortality rate was correlated with the exposure time. The rate of mortality caused by *B. subtilis* reached 64%, 74% and 77% after 24h, 48h and 72h post inoculation, respectively as compared to control, which is in the line with the present study. Similar findings were also obtained by Devi and Bora (2019), who reported that, the culture filtrates of different bacterial isolates, namely*, Bacillus* sp.*, B. thuringiensis, B. brevis, Pseudomonas* sp., *P. aeruginosa* and *P. fluorescens* significantly increased the mortality percent of *M. incognita* juveniles as compared to control. Ramezani et al. (2014) reported that isolates belonging to *Bacillus* spp. including *B. cereus* and *B. pumilus* were effective against root-knot nematodes under in vitro conditions, and culture filtrates caused juvenile mortality of 72–99% after 48 hr. The finding is in the line of present investigation. The mortality of J_2_ of root-knot nematodes may be attributed to several mechanisms when exposed to different concentrations of culture filtrates and exposure time. A number of in vitro studies reported direct antagonism by bacterial isolates towards plant-parasitic nematode species belonging to the genera *Meloidogyne, Heterodera* and *Rotylenchulus* (Gokte and Swamp, 1988; Oostendorp and Sikora, 1990; Kloepper et al., 1992; Siddiqui and Mahmood, 1995). The reduction in viability and mortality of J_2_ may be induced by production of secondary metabolites like lytic enzymes that includes gelatinase, protease and chitinase (Dunne et al., 1998; Ali et al., 2002; Mendoza et al., 2008); or the endophytic bacteria may release volatile compounds like benzene acetaldehyde 2-onanone, ecanal, 2-undecanone and dymethyl disulphide (Ali et al., 2002). Production of nematicidal compounds by rhizobacteria had been demonstrated by several workers (Becker et al., 1988; Hu et al., 1999; Li et al., 2002). Wei et al. (2003) and Qiuhong et al. (2006a, 2006b) reported the production of nematicidal compounds by *Bacillus* sp, *B. nematocide* and *B. thuringiensis*, and Niu et al. (2007) demonstrated that a serine protease in a new strain of *Bacillus* spp. played an important pathogenic factor in the control of nematodes. All these reports and the finding of present investigation indicate that the *Bacillus* group is one of the most potent bacteria that could be effectively used for the management of *M. incognita*.

Efficacy of *Bacillus* and *Pseudomonus* against *M. incognita* has been reported by various authors and is conformity with the present investigation. However, efficacy of *Microbacterium* and *Exiguobacterium* against *M. incognita* has not been reported by other authors so far.

## Conclusion

*Bacillus marisflavi, Microbacterium arborescens*, *Bacillus altitudinis* and *Exiguobacterium indicum* are noval endophytic bacteria isolated from tomato plants and can be explored as biological control agent against *Meloidogyne incognita*.
